# Hemophagocytic lymphohistiocytosis secondary to rifampin treatment: A case report

**DOI:** 10.1097/MD.0000000000039011

**Published:** 2024-07-19

**Authors:** Caihong Wang, Junke Qiu, Xiaoqing Huang, Jiekun Xu, Lei Pan

**Affiliations:** a Tuberculosis Intensive Care Unit, Zhejiang Provincial Hospital of Integrated Traditional Chinese and Western Medicine, Hangzhou, Zhejiang, China.

**Keywords:** glucocorticoids, hemophagocytic lymphohistiocytosis, rifampin, tuberculosis

## Abstract

**Rationale:**

Hemophagocytic lymphohistiocytosis (HLH) is a rare, life-threatening systemic inflammatory syndrome characterized by an overactive immune response. This hyperactivation can arise from genetic mutations, infections, malignancies, or autoimmune disorders. Medication-induced HLH is extremely rare and requires special attention.

**Patient concerns:**

A 53-year-old female diagnosed with pulmonary and urinary tract tuberculosis. She underwent quadruple therapy, including isoniazid, rifampin, ethambutol, and pyrazinamide. Subsequently, she developed fever, hepatosplenomegaly, pancytopenia, hypertriglyceridemia, hypofibrinogenemia, hyperferritinemia, increased soluble CD25 levels, decreased natural killer cell activity, and hemophagocytosis, notably without eosinophilia. Her clinical symptoms were exacerbated by rifampin intake.

**Diagnoses:**

Pulmonary and left kidney tuberculosis, multiple organ failure, and rifampin-induced HLH.

**Interventions:**

Anti-tuberculosis regimen (isoniazid, pyrazinamide, ethambutol, and levofloxacin, excluding rifampin) combined with glucocorticoid therapy.

**Outcomes:**

Satisfactory recovery with improved clinical symptoms, laboratory tests, and chest imaging studies.

**Lessons:**

Early correct diagnosis and appropriate management of HLH are essential to save the lives of affected patients. The potential severe side effects of rifampin should not be ignored.

## 1. Introduction

Hemophagocytic lymphohistiocytosis (HLH) is a rare but potentially fatal systemic inflammatory syndrome.^[1]^ It is characterized by a rapidly progressive hyperinflammatory status with excessive activations of macrophages, cytotoxic T cells, and natural killer (NK) cells, as well as destructions of erythrocytes, white blood cells, and platelets. Affected patients can have high fever, pancytopenia, hepatosplenomegaly, hypertriglyceridemia, hypofibrinogenemia, hyperferritinemia, and hemophagocytosis. Prompt diagnosis and treatments are essential to save lives.

The etiology of HLH can be classified into primary and secondary. The primary HLH is an autosomal recessive disorder due to inherited genetic mutations that are commonly present in young pediatric patients. The secondary HLH is an inappropriate overreaction of the host immune system to infection, malignancy, or autoimmune disorders.^[2]^ Among infection causes, tuberculosis can trigger HLH.^[3]^ In addition, medications could occasionally induce HLH.^[4,5]^ However, antituberculosis medication was rarely reported to cause HLH.^[6]^ Here, we report a patient who received quadruple therapy, including rifampin, due to tuberculosis. Her condition deteriorated, and she developed HLH, which worsened after resuming rifampin and improved after discontinuing rifampin and starting glucocorticoids. Finally, we diagnosed her as rifampin-induced HLH. We describe this case here and share our experience with this scarce clinical situation.

## 2. Case presentation

**Figure 1.**
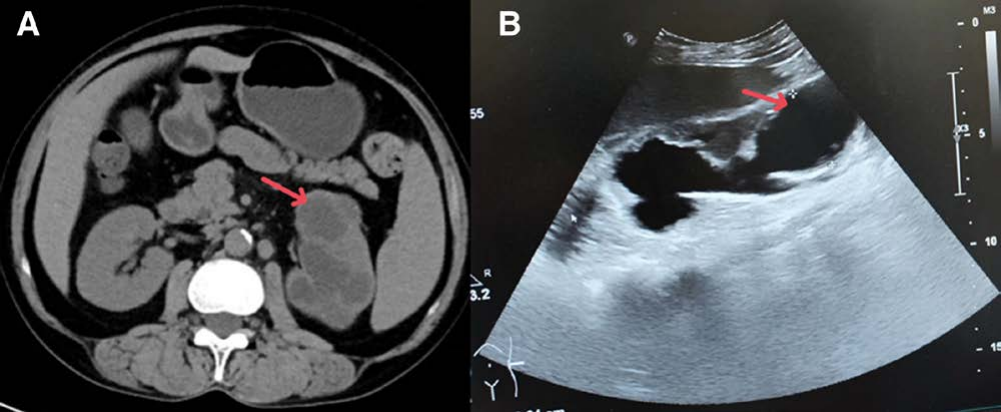
(A) Initial abdominal computed tomography scan showed low-density shadows in the left kidney. (B) At the intensive care unit admission, ultrasound showed left hydronephrosis, parenchymal atrophy, and hydroureter.

**Figure 2.**
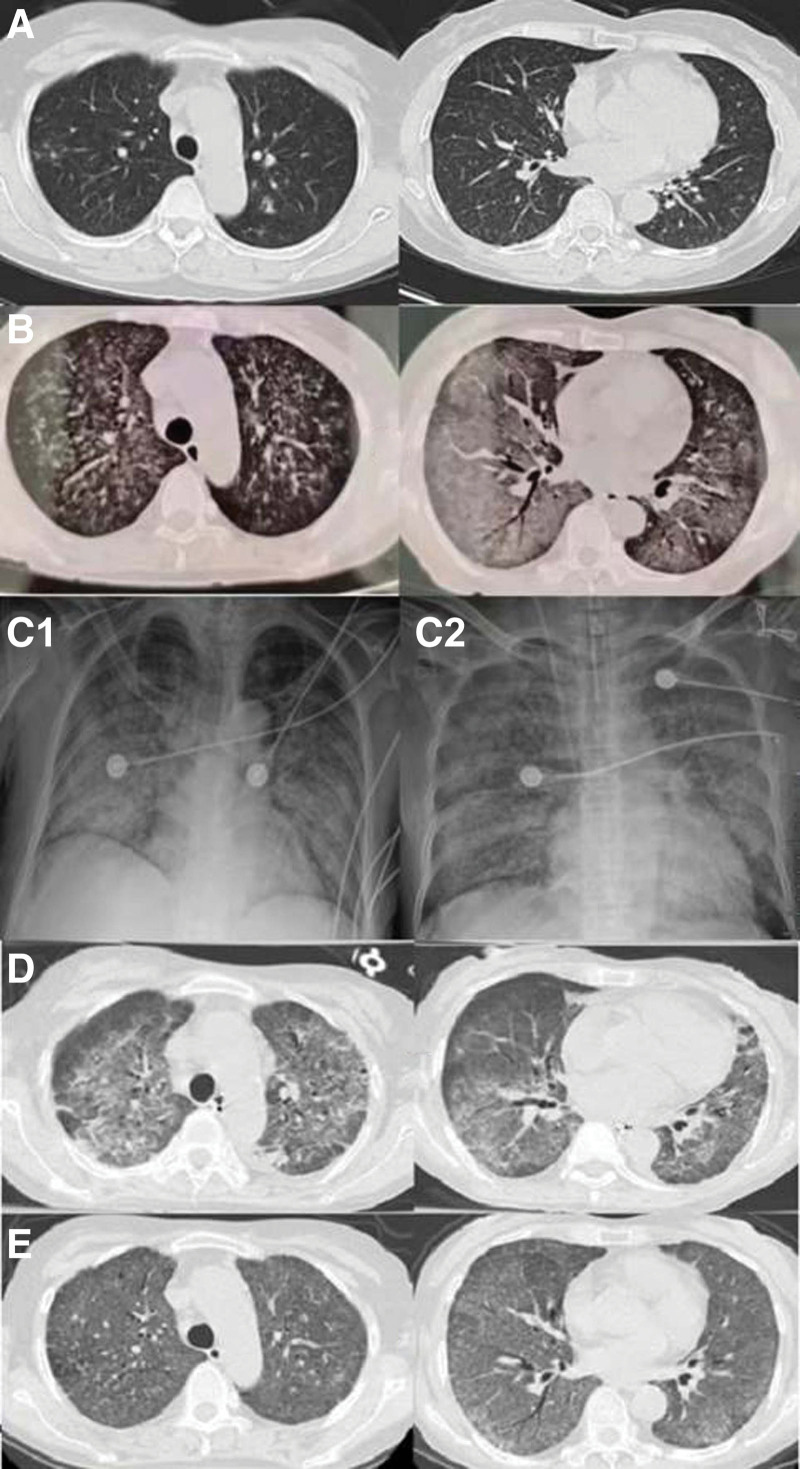
Chest computed tomography scan. (A) Bilateral pulmonary nodules during the first clinic visit. (B) Bilateral patchy infiltrations, consolidations, and ground-glass shadows 19 days after initiating antituberculosis therapy. (C) Right, at the intensive care unit admission, chest X-ray showed bilateral diffuse infiltrations. Left, after rifampin infusion on day 9, increased bilateral infiltrations. (D) Decreased bilateral lung infiltrations on day 20. (E) Improved bilateral lung infiltrations 1 month after the hospital discharge.

**Figure 3.**
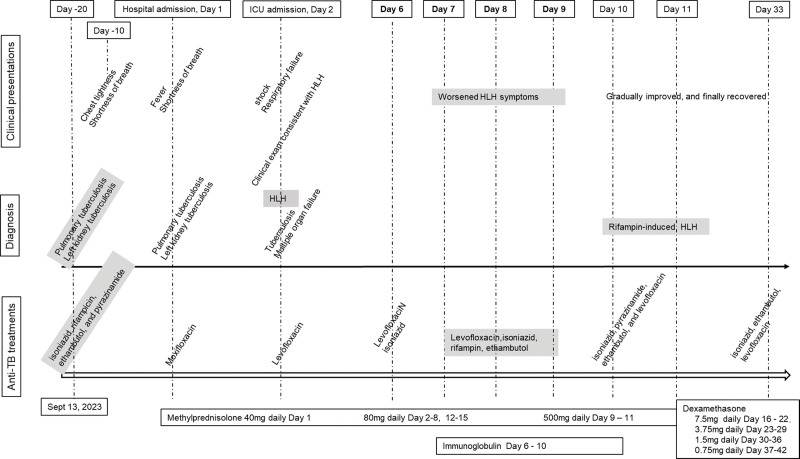
Clinical presentations, diagnosis, and treatments. The patient was initially diagnosed as pulmonary tuberculosis and left renal tuberculosis (day 20) and was started on quadruple therapy. She was admitted to hospital after symptoms worsened (day 1). Anti-tuberculosis therapy was changed to moxifloxacin. The next day (day 2), she was transferred to ICU due to multiple organ dysfunction. Anti-tuberculosis therapy was changed to levofloxacin. On day 6, she was diagnosed with hemophagocytic lymphohistiocytosis. Her conditions worsened after rifampin was added (days 7, 8, and 9) and improved after initiating high-dose methylprednisolone, and antituberculosis quadruple therapy (isoniazid, pyrazinamide, ethambutol, and levofloxacin, but without rifampin) on day 10.

A 53-year-old female presented to an external hospital on September 10, 2023, experiencing urinary frequency and urgency for over 6 months. Her medical history included abdominal angioplasty and stent placement for an aortic aneurysm, hypothyroidism treated with levothyroxine (100 μg daily), and chronic leukopenia. The physical examination revealed normal vital signs but bilateral crackles upon lung auscultation, a soft abdomen, and tenderness at the left costovertebral angle. Routine laboratory tests, such as complete blood counts, chemistry, and hepatic, and renal function assessments, were unremarkable (refer Table 1 for dynamic changes in laboratory test results). An abdominal and pelvic computed tomography (CT) scan identified low-density shadows in the left kidney (see Fig. 1) and dilation of the left renal pelvis and upper ureter. Bilateral pulmonary nodules were evident in the chest CT scan (Fig. 2A). Moreover, a 24-hour urine sample tested positive for Xpert *Mycobacterium tuberculosis*, indicating sensitivity to rifampin. She was diagnosed with secondary pulmonary tuberculosis and left renal tuberculosis. The patient was isolated and commenced antituberculosis quadruple therapy, including isoniazid, rifampin, ethambutol, and pyrazinamide, starting September 13, 2023 (Fig. 3).

**Table 1:** Dynamic changes of laboratory test results.

	Day 20	Day 10	Day 1	Day 2	Day 6	Day 7	Day 8	Day 9	Day 10	Day 11	Day 17	Day 33
Cell counts												
White blood cell, × 10^9^/L	3.5	3.2	1.6	3.8	5.6	5.6	4.6	3.9	2.6	5.9	4.8	3.8
Erythrocyte, × 10^12^/L	3.92	3.90	3.55	3.10	3.22	3.41	2.29	2.32	2.35	3.47	3.11	3.24
Platelet, × 10^9^/L	154	66	42	26	50	58	46	21	10	45	108	138
Hemoglobin, g/L	110	110	96	82	85	90	59	60	61	90	82	85
Coagulation												
Activated partial thromboplastin time, sec			-*	-*	34.7	35.5	31.1	30.9	30.9	30.2	29.8	29.0
Prothrombin time, sec			16.5	15.7	12.8	12.4	12.0	12.5	12.5	11.1	11.5	11.9
Fibrinogen, mg/dL			15	29	111	164	235	277	334	313	258	288
Hepatic functions												
Total bilirubin, µmol/L	12.6	24.2	46.7	34.4	19.7	15.4	22.2	15.4	17.0	10.7	9.2	5.1
AST, U/L	34	71	83	41	31	27	21	11	11	40	30	35
ALT, U/L	23	26	30	28	19	16	14	10	10	18	15	18
Renal functions												
Creatinine, µmol/L	94	107	207	191	160	141	143	139	125	85.3	70	66.9
BUN, µmol/L	6.2	7.2	17.3	16.3	14.8	14.1	14.2	13.9	13.3	12.2	6.7	5.8
GFR, ml/min				20								
Triglyceride, mmol/L			6.29	5.9	3.1			4.28			3.0	2.58
Ferritin, µg/L				558	338			646			449	335
Soluble CD25				32,684								

ALT = alanine transaminase, AST = aspartate transaminase, BUN = blood urine nitrogen, GFR = Glomerular filtration rate.

* Undetectable.

**Figure 4.**
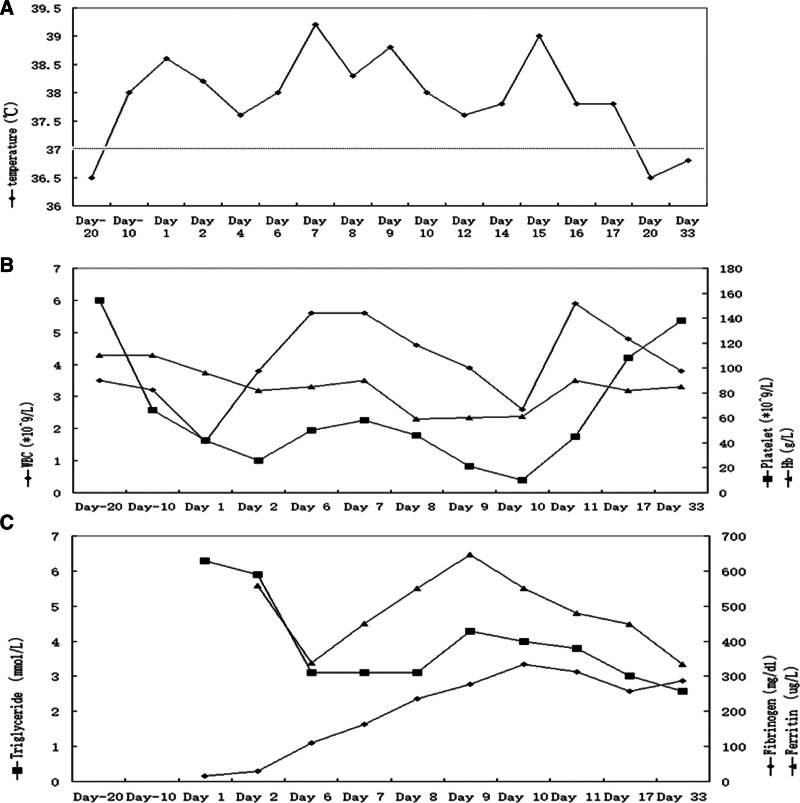
Dynamic changes in temperature (A), cell counts and hemoglobin level (B), and triglyceride, fibrinogen, and ferritin (C) during the disease course.

Nine days after initiating antituberculosis treatment (September 22, 2023), the patient reported that her urinary frequency and urgency had improved. However, she started to have a fever and exertional shortness of breath (dynamic change of temperature during the disease course is shown in Fig. 4A). Repeat laboratory tests showed slightly elevated serum creatinine (107 μmol/L) and total bilirubin (24.2 μmol/L) levels, with thrombocytopenia (66 × 10^9^/L) (dynamic changes in white blood cell count, hemoglobin level, and platelet count are shown in Fig. 4B). The same antituberculosis treatment was continued. On October 2, 2023, the patient reported progressive worsening shortness of breath and hemoptysis. A repeat chest CT scan reported significant bilateral upper lobe patchy infiltrations, middle and lower lobe consolidations, and ground-glass shadows (Fig. 2B). She was transferred to our hospital for further management the next day.

At the hospital admission on October 3, 2023, the patient’s vital signs were temperature 38.6 °C, pulse 102 beats/min, respiratory rate 28 breaths/min, and blood pressure 110/75 mm Hg. She was awake but lethargic. There were cyanosis, bilateral leg edema, and decreased breath sounds and crackles in both lungs. The laboratory test showed further increased creatinine (206 μmol/L) and total bilirubin (46.7 μmol/L) levels with leukopenia (1.6 × 10^9^/L) and thrombocytopenia (42 × 10^9^/L). The plasma fibrinogen was also decreased (15 mg/dL). Dynamic change of fibrinogen is shown in Figure 4C, and prothrombin time was prolonged (23.6 s). The arterial blood gas analysis showed a PaO_2_/FiO_2_ 206.

Following hospital admission, the patient received oxygen therapy via a face mask to maintain her oxygen saturation (SatO_2_) above 90%. Her symptoms, suspected to be adverse reactions to the antituberculosis regimen, prompted the cessation of the initial treatment. She was then started on moxifloxacin. Additionally, methylprednisolone (40 mg daily) was administered to manage potential drug-related adverse reactions. Despite these interventions, her condition deteriorated, characterized by progressively worsening breathing difficulty and edema. Laboratory tests revealed elevated serum creatinine, total bilirubin, triglycerides, lactate, and signs of pancytopenia (Fig. 4). Further abnormalities included prolonged prothrombin time, undetectable activated partial thromboplastin time, low fibrinogen levels (29 mg/dL), and elevated D-dimer, ferritin, and IL-6 levels. Her IgG, IgA, and IgM immunoglobulin levels were normal, but there was an increased CD4/CD8 ratio. Tests for autoimmune antibodies and tumor markers yielded negative results. The arterial blood gas analysis showed a decreasing PaO_2_/FiO_2_ ratio, reaching 136. Her blood pressure was recorded at 85/45 mm Hg, with a heart rate of 120 beats/min. Procalcitonin and C-reactive protein levels remained normal. The patient was diagnosed with pulmonary tuberculosis, left renal tuberculosis, shock, and acute respiratory distress. Consequently, she was transferred to the tuberculosis intensive care unit the following day (Fig. 3).

**Figure 5.**
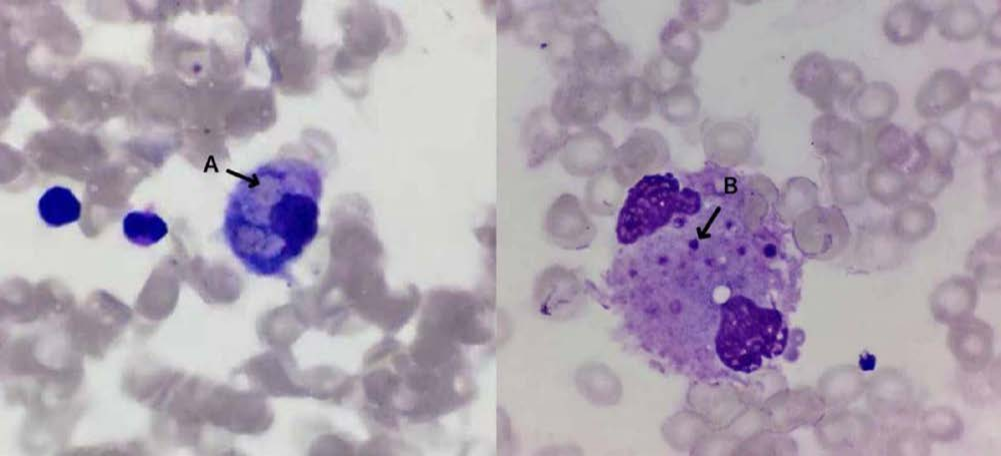
Bone marrow aspiration and biopsy showed macrophages with engulfed erythrocytes (A) and platelets (B).

In the intensive care unit, she received tracheal intubation and mechanical ventilation. There was a significant amount of light-blooded foamy secretion aspirated from the airway. Her blood pressure was maintained above the mean arterial pressure of 65 mm Hg by intravenous infusion of norepinephrine 0.63 μg/kg/min. The echocardiography was unremarkable. The abdominal ultrasound examination reported little ascites, mild hepatosplenomegaly, severe left hydronephrosis with parenchymal atrophy, and left hydroureter (Fig. 1B). The bedside chest X-ray showed bilateral diffuse infiltrations, more in the middle and lower lobes (Fig. 2C right). A bronchoscopy was performed, which showed congested bronchial mucosa with light-blooded foamy secretion. Second-generation sequencing of the alveolar lavage fluid revealed 566 sequences of *M tuberculosis*, 89 sequences of *Burkholderia cepacian*. The sputum Xpert test and *M tuberculosis* DNA were positive, but acid-fast bacilli and mycobacterial culture were negative. Increased soluble CD25 (32.684 pg/mL) and decreased NK cell activity (13.4%) were reported. The bone marrow aspiration and biopsy were performed, which showed hemophagocytosis (Fig. 5), with normal hyperplasia of bone marrow tissue and negative for acid-fast bacilli, granuloma, and malignant cells. The blood Epstein–Barr virus-DNA and cytomegalovirus-DNA were negative by the polymerase chain reaction. There was no eosinophilia. Considering her clinical presentations and testing results, we diagnosed HLH (Fig. 3). The patient received meropenem, antituberculosis with levofloxacin (0.5 g daily), methylprednisolone (40 mg, twice a day), recombinant human granulocyte stimulating factor, erythropoietin, thrombopoietin, diuresis, plasma transfusion, and supportive care.

The patient’s temperature gradually decreased to 37.6 °C, and there was an improvement in her shortness of breath. Laboratory test results also showed signs of improvement. Given her diagnosis of tuberculosis, we initially considered her HLH to be secondary to this condition. On the sixth day of her hospital stay, isoniazid was reintroduced into her antituberculosis treatment, along with intravenous immune globulin. Rifampin and ethambutol were added on day 7. However, approximately half an hour following the intravenous infusion of rifampin, the patient developed a high fever and worsening shortness of breath, with a PaO_2_/FiO_2_ ratio of 136. She exhibited an increase in bloody foamy secretion from airway aspiration. Her blood pressure fell to 55/35 mm Hg, her heart rate decreased from 120 to 75 beats/min, and her SatO_2_ dropped to 78%. The patient underwent resuscitation with 100% oxygen and a combination of epinephrine and norepinephrine to maintain her blood pressure. Intravenous methylprednisolone (40 mg) was administered. Despite these interventions, rifampin was continued on days 8 and 9. We concluded that a moderate dose of steroids (methylprednisolone 40 mg twice a day) was insufficient to control the symptoms of HLH.

Consequently, on day 9, the patient was given a higher dose of intravenous methylprednisolone at 500 mg daily for 3 days. She continued to experience persistent fever, a PaO_2_/FiO_2_ ratio of 136, worsening pancytopenia, increased bilirubin and triglyceride levels, heightened airway secretions, and the development of petechiae in her lower extremities. A repeat bedside chest X-ray showed increased bilateral infiltrations extending to the bilateral upper lobes (Fig. 2C, right). Suspecting drug-induced HLH potentially triggered by rifampin, we discontinued the drug on day 10. The antituberculosis regimen was modified to include isoniazid, pyrazinamide, ethambutol, and levofloxacin. The vasopressor was stopped the next day. However, her symptoms worsened after the methylprednisolone dosage was reduced back to 40 mg twice a day.

Then, we followed the HLH-94 protocol and switched to dexamethasone (7.5 mg daily) on day 16 (Fig. 3). The patient’s condition gradually improved, and she was extubated on day 18. Repeat laboratory tests showed gradually improved cell counts, hepatic and renal functions, triglycerides, and inflammatory markers. The chest CT scan showed decreased bilateral infiltrations on day 20 (Fig. 2D). The patient was transferred to the medical floor on day 22. Pyrazinamide was discontinued due to extreme hyperuricemia (705 µmol/L) on day 30, and she was discharged from the hospital on day 33. One month later, the patient reported no cough or shortness of breath during the outpatient follow-up visit. Her vital signs and laboratory tests were within normal limits. A repeat chest CT scan shows significantly improved bilateral lung infiltrations (Fig. 2E). The antituberculosis therapy was scheduled for a total of 12 months for her.

## 3. Discussion

HLH is an over-reacted immune system disorder due to various primary or secondary causes. Primary HLH usually happens in children. Secondary HLH can be caused by infection, malignancy, or autoimmune diseases. Previous studies have reported that tuberculosis infection could cause HLH.^[3]^ Our patient had pulmonary and urinary tract tuberculosis. Her HLH developed after the initiation of antituberculosis treatment. Her examinations revealed fever, pancytopenia, hypertriglyceridemia, hepatosplenomegaly, hemophagocytosis, and increased inflammatory markers, which was consistent with the diagnosis of HLH. Rifampin administration could exacerbate her symptoms. Discontinuing rifampin treatment and the addition of steroids could relieve her symptoms. Therefore, we believe that the HLH in this patient was induced by rifampin, which was never reported previously. We want to discuss this case to remind the clinicians about this rare situation.

The typical findings in HLH included fever, cytopenia, splenomegaly, hypertriglyceridemia, hemophagocytosis, low or absent NK cells, and increased inflammatory markers, including ferritin, soluble CD25, and CXCL9.^[1]^ There were many cases of tuberculosis-associated HLH reported previously. Both HLH and tuberculosis can involve multiple organ systems, leading to multi-organ failure and death. However, their treatments were distinctly different. This dramatically challenges clinicians to make prompt and accurate diagnoses.

Tuberculosis is caused by *M tuberculosis*. The lung is the most commonly involved organ in patients with tuberculosis. Affected patients can have fever, cough, shortness of breath, and respiratory failure. In addition, extrapulmonary tuberculosis can happen to multiple organ systems, such as gastrointestinal, genitourinary, and musculoskeletal systems, to have related symptoms.^[7]^ The diagnosis of tuberculosis depends on identifying *M tuberculosis* in the sputum, blood, urine, body fluid, or tissue specimens. Its treatment commonly requires a combination of several antituberculosis medications to shorten the treatment duration, lower the resistance rate, and decrease the chance of relapse. Our patient had pulmonary and urinary tract tuberculosis. The diagnosis of tuberculosis was established based on the positive Xpert test on the sputum and urine specimens of this patient. The Xpert is a nucleic acid amplification test with high specificity and sensitivity. It also reports bacterial sensitivity to rifampin.^[8]^ The tuberculosis in our patient was sensitive to rifampin. Therefore, an antituberculosis regimen, including isoniazid, rifampin, ethambutol, and pyrazinamide, was initiated and prescribed to her. However, instead of improving, her symptoms deteriorated. At our hospital, she presented with fever, respiratory failure, hemoptysis, and diffuse ground-glass opacities and consolidations visible in the chest CT scan. Further evaluations revealed pancytopenia, hypertriglyceridemia, hepatosplenomegaly, hemophagocytosis, and elevated inflammatory markers. These findings supported a diagnosis of HLH,^[9]^ which had not been evident before the anti-tuberculous treatment, leading us to suspect drug-induced HLH.

This suspicion finds parallels in the literature. Jain and Dash reported on a 22-year-old male with tuberculous lymphadenitis who developed fever, pancytopenia, and jaundice. A bone marrow biopsy in this case revealed extensive hemophagocytosis, and the patient was diagnosed with HLH, thought to be induced by isoniazid. Following a switch to an alternative antituberculosis regimen, he fully recovered.^[6]^ Approximately 9 days after initiating the antituberculosis regimen that included rifampin, the patient showed worsening symptoms. Her condition improved with the continuation of antituberculosis treatment using levofloxacin and the addition of steroids. However, reintroducing rifampin worsened her symptoms again, aligning with the diagnostic criteria of HLH. She ultimately achieved full recovery with a steroid regimen and an antituberculosis treatment excluding rifampin, leading us to conclude that she had rifampin-induced HLH.

Notably, about half of HLH patients can have pulmonary involvement.^[10]^ Typical lung imaging findings may include atelectasis, nodules, consolidations, and ground-glass opacities. Pleural effusion and mediastinal lymph node disease have also been reported. A lung biopsy and pathological examination can reveal signs of immune lung injury, supporting an HLH diagnosis. However, lung biopsies are not always feasible or accepted by every patient.

Rifampin inhibits DNA-dependent RNA polymerase, contributing to its antimicrobial activity. Patients on rifampin may experience orange discoloration of body fluids, such as urine, sweat, and saliva. Common side effects include gastrointestinal symptoms and hepatotoxicity.^[11]^ Re-exposure to rifampin can lead to hypersensitive reactions like rash, urticaria, flu-like symptoms, shock, or multi-organ failure.^[12]^ In this report, we highlight another severe reaction, HLH, following rifampin administration.

The diagnosis of HLH necessitates the exclusion of drug reaction with eosinophilia and systemic symptoms, a severe drug reaction characterized by fever, rash, and multiple organ involvement. In drug reaction with eosinophilia and systemic symptom cases, blood tests typically reveal eosinophilia and leukocytosis, contrasting with the pancytopenia observed in our patient. This distinction is crucial for accurate diagnosis and treatment planning.

For patients diagnosed with HLH, the HLH-94 treatment protocol is recommended to suppress life-threatening inflammatory reactions.^[13]^ This protocol incorporates dexamethasone and etoposide over 8 weeks. In the case of our patient, who had concurrent tuberculosis and HLH, we modified this approach. Given her active tuberculosis infection and impaired hepatic and renal functions, we chose not to use etoposide. Instead, we relied solely on steroids and antituberculosis therapy, which ultimately yielded successful outcomes. It is noteworthy that our patient’s symptoms temporarily worsened upon reducing the steroid dosage, likely due to incomplete mitigation of the hyperactive inflammatory response driven by cytokines. Beginning in week 3, we transitioned to dexamethasone as per the HLH-94 protocol, eventually achieving satisfactory results. We advocate that severe drug-induced HLH necessitates high-dose pulse corticosteroid therapy and subsequent tapering with dexamethasone maintenance therapy for 6 to 8 weeks, along with discontinuation of the offending drug, to ensure complete resolution.

## 4. Conclusions

In conclusion, this case report details a patient who developed HLH during antituberculosis treatment attributed to rifampin. A revised antituberculosis regimen, excluding rifampin, combined with steroid therapy, facilitated her full recovery. Clinicians managing similar cases should remain vigilant for drug-induced HLH and adjust treatment regimens accordingly.

## Author contributions

**Conceptualization:** Caihong Wang.

**Formal analysis:** Junke Qiu.

**Funding acquisition:** Xiaoqing Huang, Jiekun Xu, Lei Pan.

**Project administration:** Junke Qiu, Xiaoqing Huang, Jiekun Xu.

**Resources:** Junke Qiu, Lei Pan.

**Validation:** Caihong Wang.

**Visualization:** Xiaoqing Huang, Jiekun Xu.

**Writing – original draft:** Caihong Wang, Junke Qiu, Xiaoqing Huang, Jiekun Xu, Lei Pan.
